# Novel Volumetric and Surface-Based Magnetic Resonance Indices of the Aging Brain – Does Male and Female Brain Age in the Same Way?

**DOI:** 10.3389/fneur.2021.645729

**Published:** 2021-06-07

**Authors:** Przemysław Podgórski, Joanna Bladowska, Marek Sasiadek, Anna Zimny

**Affiliations:** Department of General and Interventional Radiology and Neuroradiology, Wroclaw Medical University, Wrocław, Poland

**Keywords:** brain volumetry, cortical thickness, sulcal depth, gyrification index, fractal dimension, brain aging, region-based morphometry, surface-based morphometry

## Abstract

**Introduction:** Novel post-processing methods allow not only for assessment of brain volumetry or cortical thickness based on magnetic resonance imaging (MRI) but also for more detailed analysis of cortical shape and complexity using parameters such as sulcal depth, gyrification index, or fractal dimension. The aim of this study was to analyze changes in brain volumetry and other cortical indices during aging in men and women.

**Material and Methods:** Material consisted of 697 healthy volunteers (aged 38–80 years; M/F, 264/443) who underwent brain MRI using a 1.5-T scanner. Voxel-based volumetry of total gray matter (GM), white matter (WM), and cerebrospinal fluid (CSF) was performed followed by assessment of cortical parameters [cortical thickness (CT), sulcal depth (SD), gyrification index (GI), and fractal dimension (FD)] in 150 atlas locations using surface-based morphometry with a region-based approach. All parameters were compared among seven age groups (grouped every 5 years) separately for men and women. Additionally, percentile curves for men and women were provided for total volumes of GM, WM, and CSF.

**Results:** In men and women, a decrease in GM and WM volumes and an increase in CSF volume seem to progress slowly since the age of 45. In men, significant GM and WM loss as well as CSF increase start above 55 years of age, while in women, significant GM loss starts above 50 and significant WM loss as well as CSF increase above 60. CT was found to significantly decrease with aging in 39% of locations in women and in 36% of locations in men, SD was found to increase in 13.5% of locations in women and in 1.3% of locations in men, GI was decreased in 3.4% of locations in women and in 2.0% of locations in men, and FD was changed in 2.7% of locations in women compared to 2.0% in men.

**Conclusions:** Male and female brains start aging at the similar age of 45. Compared to men, in women, the cortex is affected earlier and in the more complex pattern regarding not only cortical loss but also other alterations within the cortical shape, with relatively longer sparing of WM volume.

## Introduction

Brain aging is a physiological process occurring with age leading to atrophy of different structures. Brain volumetry using magnetic resonance (MR) data has been used for many years allowing for *in vivo* assessment of the size of brain structures. The most commonly applied methods are region-based volumetry (RBM) or voxel-based morphometry (VBM) (1). They are widely used to get insight into processes of both physiological and pathological aging. The knowledge on normal processes during brain aging is crucial to better understand changes in the course of specific diseases especially leading to brain degeneration such as Alzheimer's disease and other dementias, Parkinson disease, or multiple sclerosis (2–5).

Novel methods of post-processing of structural MR data allow for assessment of not only the volume of brain structures but also cortical thickness (CT) or even for more detailed analysis of the cortex shape and complexity including parameters such as sulcal depth (SD), gyrification index (GI), or fractal dimension (FD). Cortical thickness mapping provides information about neuronal loss or degradation indicated by thinning of the cortex. The CT of the human brain is approximately 1–4.5 mm depending on the brain region with mean of 2.5 mm (6, 7). The SD and GI are parameters referring to cortical folding, which is remarkably related to brain connectivity and functional development (8). The GI is a metric that quantifies the amount of cortex buried within the sulcal folds as compared with the amount of cortex on the outer visible cortex. A cortex with extensive folding has a high GI, whereas a cortex with limited folding shows lower GI (9). On the other hand, FD is a morphometric measure that has been used to investigate cortical folding complexity. Fractal dimension has been found to correlate with IQ and the number of years of education; thus, it has a significant relationship with intelligence and education (10). Cortical indices such as CT, GI, SC, and FS have been reported to change in older people and in neurodegenerative diseases (11, 12). In healthy adults, age-related thinning is observed across the majority of the cortex but is generally more robust in bilateral frontal cortices, superior temporal regions, and supramarginal gyri (13). Several studies have shown that global cortical gyrification reflected by GI decreases with age in several cross-sectional samples, and topography of these changes is different from CT (14, 15). Age relates to lower gyrification mainly in the parietal, frontal, temporal, and occipital regions (16). Using quantitative approaches, SD has been shown to increase with age as well as to be associated with decreases in cognitive abilities and physical activity (14). Being a relatively new concept of analyzing brain complexity, FD has been reported only in few studies to be reduced in the course of aging process without a linear correlation with the results of CT or GI (17, 18).

Aging of the brain is a complex process affecting the cerebral tissue, vasculature, and cognition at all levels from molecules, gross morphology to function. It usually starts in the 30–40 years of age with the reduction in brain volume at a rate of 5% per decade after 40 years of age. Brain aging is influenced by many factors including genetic, environmental, or coexisting diseases (19, 20). It has been postulated that sex differences may influence brain morphology and physiology during both development and aging (21, 22). Some studies indicate that the male brain may age faster than the female brain. It has already been reported that the brains of women are more resilient to cognitive decline, with older women tending to score higher in tests of reason, memory, and problem solving than men of the same age (23, 24). Sex differences during brain development likely set the stage for brain aging later in life. Differences in hormones levels, cerebral blood flow, and gene expression may make the female brain more resilient to aging-related stressors (25). Goyal et al. found that throughout the adult life span, the female brains have a persistently lower metabolic brain age relative to their chronological age-compared male brains (26). Although anatomical sex-related differences in brain volumetry have already been published, complexity of the cortex and cortical aging have not been thoroughly characterized with respect to sex differences in the human brain.

The aim of this study was to analyze changes in brain volumetry and other cortical indices during aging separately in men and women. To our knowledge, there have been only few reports on differences in cortical parameters between male and female brains, but as far as we know, there are no papers focusing on detailed patterns of cortical aging in both sexes on the basis of several evaluated cortical parameters derived from VBM and surface-based morphometry (SBM) with the application of a region-based volumetry (RBV) approach. This study looks at these patterns and gives detailed insight into changes in several cortical parameters such as CT, GI, SD, and FD in 150 atlas locations separately in men and women between 38 and 80 years of age in a Central Europe Caucasian population. To our knowledge, this is the first article evaluating so many different cortical parameters during aging separately in men and women in a large cohort of subjects using exactly the same scanning protocol and post-processing method. We believe that knowledge about the complexity of sex-specific brain aging is crucial before the extent evaluation of pathological brain atrophy in different neurological and psychiatric disorders. Increased understanding of the neurobiology of sex-based differences in brain variability across the lifespan can provide insight into both disease vulnerability and resilience.

## Materials and Methods

The study was supported by Wroclaw Medical University Grant SUB.C270.21.020 and conducted in accordance with the guidelines of the Wroclaw Medical University Ethics Committee for conducting research involving humans permission No. KB-591/2019. Each participant signed an informed consent prior to the study enrolment.

Material consisted of 697 healthy volunteers (male/female, 264/443) with age ranging between 38 and 80 years (Table 1) who underwent structural brain MRI using a 1.5-T scanner (Signa HDxt, GE Healthcare) with an eight-channel head coil. The scanning protocol included T2, fluid-attenuated inversion recovery (FLAIR), diffusion-weighted imaging (DWI), and susceptibility weighted imaging (SWI) sequences as well as a T1-3D inversion-recovery-prepared fast spoiled gradient recalled (IR FSPGR) volumetric sequence [field of view (FOV), 24 × 24 cm; matrix, 240 × 240; voxel size, 1 × 1 × 1 mm; flip angle, 12; repetition time (TR) = 8.34; echo time (TE) = 3.192]. Study exclusion criteria were as follows: history of stroke or other neurological or psychiatric diseases including cognitive impairment, as well as diabetes, hypertension, hypercholesterolemia, symptomatic cardiovascular disease, or any other chronic diseases requiring medications, abnormal body mass index (BMI), substance abuse, and contraindications to MRI. Patients were assessed with the Mini-Mental State Examination (MMSE), and their score corrected for age and education level resulted within the normal range both in men and women (Table 1). T2-weighted and FLAIR images were evaluated for signs of severe cerebral small vessel disease and subjects with high volumes of white matter hyperintensities (WMHs) graded with Fazekas score 2–3; lacunar infarcts or microbleeds were also excluded from the study.

**Table 1:** Distribution of men and women as well as their Mini-Mental State Examination (MMSE) scores in different age groups.

**Age groups in years**	**Number of men**	**Mean MMSE score in men**	**Number of women**	**Mean MMSE score in women**
>45	22	30	26	30
46–50	37	30	34	30
51–55	31	30	58	30
56–60	42	29	95	29.5
61–65	54	28.5	113	28.0
66–70	43	28.0	63	28.5
71–75	22	27.5	32	28.0
76–80	13	26.5	12	27.0
Total	264	28.7	443	28.9

Data processing workflow comprised of two steps: voxel- and surface-based processing assessed using the Computational Anatomy Toolbox 12 (CAT12, Structural Brain Imaging Group, University of Jena) and the Statistical Parametric Maps 12 (SPM12) software with the application of RBM approach. The initial voxel processing steps included a spatial adaptive non-local means (SANLM) denoising filter (27); then, data were bias corrected and affine registered followed by the standard SPM “unified segmentation” (28). Further steps included skull stripping of the brain, parcellation into the left and right hemisphere, subcortical areas, and the cerebellum. To address the problem of gray matter intensities, a local intensity transformation of all tissue classes was performed. Final steps included adaptive maximum *a posteriori* (AMAP) segmentation (29), which was then refined by applying a partial volume estimation (30). As the last default step, the tissue segments were spatially normalized to a common reference space using DARTEL (31). Surface-based processing included all forementioned steps and registration of the cortical surfaces of the two hemispheres to the FreeSurfer template. As a final step, calculation of region of interest (ROI)-based measures was performed for voxel- and surface-based indices. For quality assurance, a two-step process was used. First, before preprocessing in CAT12, overall data quality was checked, and datasets with artifacts were rejected. Second, the quality control measures incorporated in the CAT12 processing pipeline were used to identify the corrupted data after segmentation. All data used in the final analysis scored at CAT12 interquartile range (IQR) from 84 to 96%. These data were analyzed further for the presence of newly introduced artifacts. Of 730 initially scanned subjects, 33 were rejected due to incomplete or insufficient data quality resulting from movement artifacts or aborted scanning. Finally, 697 subjects were included in the study.

Volumetric measurements consisted of the volumes of the total gray matter (GM), total white matter (WM), and total cerebrospinal fluid (CSF), while the cortical surface parameters such as CT, SD, GI, and FD were assessed in 150 cortical atlas locations developed by Destrieux (h.aparc.a2009s.annot) (32). All evaluated parameters were compared among seven age groups (below 45 years of age and then grouped every 5 years up to 80 years) separately for men and women using R software. Multiple comparisons of the volumes of total GM, WM, and CSF among the age groups were performed using Kruskal–Wallis test with the significant p value set at a very strict level of below 0.0000001 followed by the *post-hoc* test with significant *p*-values set below 0.05. Additionally, percentile curves were provided for the total volumes of GM, WM, and CSF separately for men and women using GAMLSS in R. Cortical parameters such CT, GI, SD, and FD were compared between the age groups using Kruskal–Wallis test without a *post-hoc* test. In this comparison, only the results with a very low *p* ≤ 0.0001 were regarded as significant.

## Results

**Figure 1:**
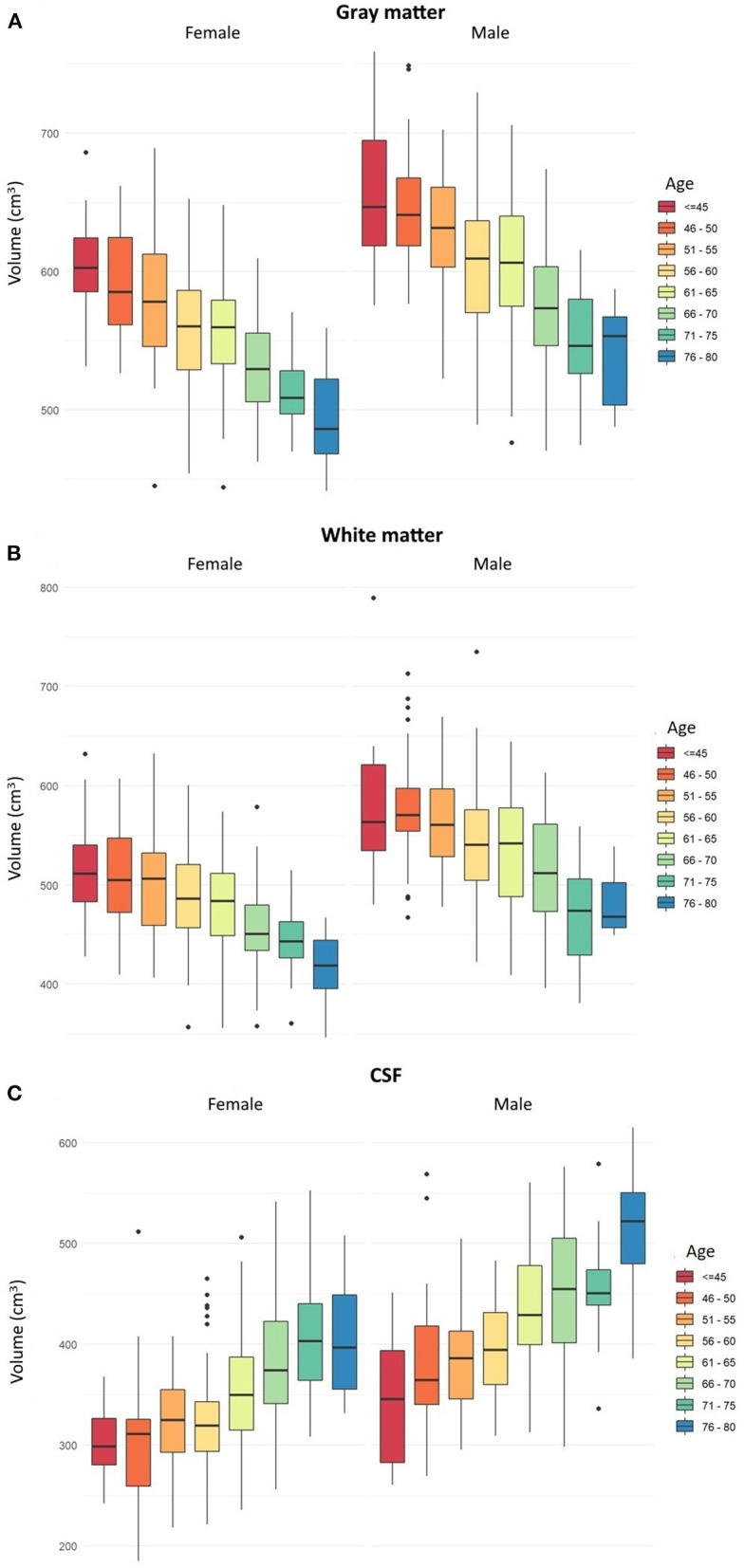
Box plots showing age-related changes in the volumes of **(A)** total gray matter, **(B)** total white matter, and **(C)** cerebrospinal fluid separately in men and women. Error bars show the standard error of the mean values. Dots represent single subjects with outlier values. The box plots a, b and c are based on parametric information and statistical analyses from Tables 1–3, respectively.

In each age group, the mean volumes of total GM, total WM, and total CSF were significantly lower in women compared to men (Tables 2–4, Figure 1). Mean volume of GM for all age groups in men was 599.58 cm^3^, while in women, 553.9 cm^3^ (8% difference). Mean volume of WM for all age groups in men was 532.9 cm^3^, while in women, 475.8 cm^3^ (11% difference). Mean volume of CSF for all age groups in men was 420 cm^3^, while in women, 349.8 cm^3^ (17% difference).

**Table 2:** The results of total gray matter measurements in different age groups and comparisons of the gray matter volumes among the age groups separately in men and women.

**Gray matter volume**								
**Male**								
**Age**	**Number**	**Mean**	**SD**	**Min**	**Q1**	**Median**	**Q3**	**Max**
≤ 45	22	653.4	50.5	575.6	618.6	646.8	694.7	758.8
46–50	37	647	38.48	576.6	618.4	641.1	667.4	748.8
51–55	31	629.5	40.98	522.7	603.2	631.5	660.7	702.5
56–60	42	603.5	54.17	489.5	570.4	609.3	636.7	729.3
61–65	54	604.5	54.11	476.2	575	606.5	640.1	705.8
66–70	43	572.2	43.17	470.6	546.4	573.6	603.5	673.8
71–75	22	548.5	40.7	474.7	526.2	546.4	580	615.5
76–80	13	538.1	36.34	487.6	503.3	553.4	567.1	587.4
**Kruskal–Wallis chi-squared = 99, df = 7**, ***p*** **< 0.0000000000000002**								
**Age**	**≤45**	**46–50**	**51–55**	**56–60**	**61–65**	**66–70**	**71–75**	
46–50	0.92							
51–55	0.27	0.26						
56–60	0.003^*^	0.001^*^	0.05					
61–65	0.003^*^	0.0008^*^	0.05	0.92				
66–70	0.0000^*^	0.0000^*^	0.0000^*^	0.007^*^	0.003^*^			
71–75	0.0000^*^	0.0000^*^	0.0000^*^	0.0004^*^	0.0002^*^	0.19		
76–80	0.0000^*^	0.0000^*^	0.0000^*^	0.0006^*^	0.0004^*^	0.11	0.68	
**Female**								
**Age**	**Number**	**Mean**	**SD**	**Min**	**Q1**	**Median**	**Q3**	**Max**
≤ 45	26	604.5	35.12	531.7	585.4	602.7	624.3	686.1
46–50	34	590.4	38.2	526.7	561.4	585.3	624.4	662
51–55	58	581	45.66	445.1	545.7	578.1	612.5	689
56–60	95	560.1	41.22	454.3	528.9	560.6	586.3	652.4
61–65	113	556.6	35.04	443.9	533.2	559.8	579.2	648
66–70	63	531.3	33.63	462.6	505.8	529.7	555.3	609.3
71–75	32	513.4	23.73	469.8	497.2	508.7	528.3	570.4
76–80	12	494.2	36.7	441.3	468.3	486.2	522.3	559
**Kruskal–Wallis chi-squared = 138, df = 7**, ***p*** **< 0.0000000000000002**								
**Age**	**≤45**	**46–50**	**51–55**	**56–60**	**61–65**	**66–70**	**71–75**	
46–50	0.27							
51–55	0.03^*^	0.34						
56–60	0.0000^*^	0.001^*^	0.01^*^					
61–65	0.0000^*^	0.0005^*^	0.003^*^	0.69				
66–70	0.0000^*^	0.0000^*^	0.0000^*^	0.0001^*^	0.0001^*^			
71–75	0.0000^*^	0.0000^*^	0.0000^*^	0.0000^*^	0.0000^*^	0.06		
76–80	0.0000^*^	0.0000^*^	0.0000^*^	0.0000^*^	0.0001^*^	0.06	0.57	

*SD, standard deviation; min, minimum value; q1, first quartile; q3, third quartile; max, maximum value*.

* *Statistically significant p-values below 0.05*.

In both men and women, a decrease in the GM and WM volumes and an increase in the CSF volume were found to progress gradually since the age below 45 years till 80. In men, the first significant GM loss, compared to younger men, was noted in the age group of 56–60 years, while in women, the first significant GM loss, compared to younger women, was revealed in the age group of 51–55. In men, significant differences in the volume of total GM were seen every 10–15 years, while in women, every 5–10 years. In both men and women, there were no significant changes in the GM volumes between different age groups above 66–70 years (Table 2 and Figure 1A).

In men, the first significant WM loss, compared to younger men, was found in the age group of 56–60, while in women, the first significant WM decrease, compared to younger women, was revealed in the age group of 61–65 years. In men, significant differences in the volume of total WM were seen every 10–15 years, while in women, different intervals of 5–15 years. In men, there were no significant changes in the volumes of total WM between different age groups above 66–70, while in women, no significant changes in WM volumes were seen in older age group above 71–75 (Table 3 and Figure 1B).

**Table 3:** The results of total white matter measurements in different age groups and comparisons of the white matter volumes among the age groups separately in men and women.

**White matter volume**								
**Male**								
**Age**	**Number**	**Mean**	**SD**	**Min**	**Q1**	**Median**	**Q3**	**Max**
≤ 45	22	575.8	67.06	480.2	534.3	563.4	621	789.3
46–50	37	576.2	56.55	467	554.2	570.6	597.1	712.9
51–55	31	564.9	47.61	477.6	528.3	560.7	596.5	669.3
56–60	42	543.4	63.66	421.8	504.3	540.8	575.7	735
61–65	54	535.6	58.15	408.7	488.3	541.8	577.4	644.6
66–70	43	516.6	53.13	396	473.1	511.8	561.2	612.9
71–75	22	470	52.71	380.9	428.9	473.8	506.1	559
76–80	13	480.9	28.81	449.2	456.6	468	502.1	538.6
**Kruskal–Wallis chi-squared = 65, df = 7**, ***p*** **= 0.00000000001**								
**Age**	**≤45**	**46–50**	**51–55**	**56–60**	**61–65**	**66–70**	**71–75**	
46–50	0.81							
51–55	0.86	0.64						
56–60	0.01^*^	0.02^*^	0.11					
61–65	0.04^*^	0.009^*^	0.06	0.81				
66–70	0.002^*^	0.0002^*^	0.002^*^	0.1	0.14			
71–75	0.0000^*^	0.0000^*^	0.0000^*^	0.0002^*^	0.0002^*^	0.06		
76–80	0.0001^*^	0.0000^*^	0.0001^*^	0.002^*^	0.002^*^	0.06	0.99	
**Female**								
**Age**	**Number**	**Mean**	**SD**	**Min**	**Q1**	**Median**	**Q3**	**Max**
≤ 45	26	512.5	47.57	427.7	482.7	511.7	540.1	632
46–50	34	507.4	48.55	409.3	472.3	504.9	546.9	607.1
51–55	58	499	51.25	406.8	458.9	506.1	532.2	632.4
56–60	95	488.6	48.23	356.3	456.6	486	520.5	600.4
61–65	113	482.5	42.91	355.4	448.7	484.1	511.4	574
66–70	63	456.5	42.02	357.2	433.9	450.4	479.5	578.2
71–75	32	442.5	31.69	360.2	426.2	443	463	514.8
76–80	12	417.7	37.44	346.1	395.4	418.7	444.1	467.1
**Kruskal–Wallis chi-squared = 82, df = 7**, ***p*** **= 0.000000000000005**								
**Age**	**≤45**	**46–50**	**51–55**	**56–60**	**61–65**	**66–70**	**71–75**	
46–50	0.75							
51–55	0.34	0.48						
56–60	0.07	0.10	0.29					
61–65	0.02^*^	0.03^*^	0.08	0.47				
66–70	0.0000^*^	0.0000^*^	0.0000^*^	0.0001^*^	0.0009^*^			
71–75	0.0000^*^	0.0000^*^	0.0000^*^	0.0000^*^	0.0000^*^	0.17		
76–80	0.0000^*^	0.0000^*^	0.0000^*^	0.0000^*^	0.0001^*^	0.04^*^	0.32	

*SD, standard deviation; min, minimum value; q1, first quartile; q3, third quartile; max, maximum value*.

* *Statistically significant p-values below 0.05*.

In men, the first significant CSF increase, compared to younger men, was detected in the age group of 56–60, while in women, in the age group of 61–65. In both men and women, significant differences in the volume of CSF were seen in different age intervals of 5–15 years. In men, there were no significant changes in the volumes of CSF between different age groups above 71–75 years, while in women, above 66–70 years (Table 4 and Figure 1C).

**Table 4:** The results of total cerebrospinal fluid (CSF) measurements in different age groups and comparisons of the CSF volumes among the age groups separately in men and women.

**CSF volume**								
**Male**								
**Age**	**Number**	**Mean**	**SD**	**Min**	**Q1**	**Median**	**Q3**	**Max**
≤ 45	22	343.4	59.02	260.5	282.6	345.7	393.4	451.1
46–50	37	379.7	64.12	269.3	340.3	364.7	417.8	568.6
51–55	31	384.3	54.81	295.4	345.5	386.4	412.7	504.5
56–60	42	396.1	44.94	309.2	359.9	394.4	431.4	482.8
61–65	54	436.1	58.38	312.6	399.7	428.8	478.0	560.0
66–70	43	450.6	72.18	298.4	401.4	454.9	505.1	575.7
71–75	22	453.1	49.81	336.0	438.7	450.4	473.5	578.4
76–80	13	516.8	67.70	385.6	479.5	522.1	549.9	615.1
**Kruskal–Wallis chi-squared = 83, df = 7**, ***p*** **= 0.000000000000003**								
**Age**	**≤45**	**46–50**	**51–55**	**56–60**	**61–65**	**66–70**	**71–75**	
46–50	0.16							
51–55	0.11	0.73						
56–60	0.01^*^	0.28	0.46					
61–65	0.0000^*^	0.0001^*^	0.001^*^	0.006^*^				
66–70	0.0000^*^	0.0000^*^	0.0001^*^	0.0009^*^	0.46			
71–75	0.0000^*^	0.0001^*^	0.0003^*^	0.001^*^	0.28	0.64		
76–80	0.0000^*^	0.0000^*^	0.0000^*^	0.0000^*^	0.007^*^	0.03^*^	0.12	
**Female**								
**Age**	**Number**	**Mean**	**SD**	**Min**	**Q1**	**Median**	**Q3**	**Max**
≤ 45	26	301.9	32.95	241.9	280.5	298.6	326.3	367.8
46–50	34	302.2	65.22	184.8	258.9	311.2	325.6	511.4
51–55	58	323.1	41.18	218.0	292.8	325.2	354.8	407.9
56–60	95	323.5	46.79	221.3	293.6	319.2	343.0	464.7
61–65	113	355.5	61.47	235.6	314.6	349.6	387.3	620.6
66–70	63	378.5	61.15	255.9	341.0	374.3	422.5	541.5
71–75	32	404.6	57.81	308.5	364.2	403.1	440.1	552.2
76–80	12	409.3	58.67	331.3	355.4	396.7	448.7	507.9
**Kruskal–Wallis chi-squared = 114, df = 7**, ***p*** **< 0.0000000000000002**								
**Age**	**≤45**	**46–50**	**51–55**	**56–60**	**61–65**	**66–70**	**71–75**	
46–50	0.73							
51–55	0.07	0.13						
56–60	0.08	0.14	0.83					
61–65	0.0000^*^	0.0000^*^	0.002^*^	0.0002^*^				
66–70	0.0000^*^	0.0000^*^	0.0000^*^	0.0000^*^	0.01^*^			
71–75	0.0000^*^	0.0000^*^	0.0000^*^	0.0000^*^	0.0003^*^	0.13		
76–80	0.0000^*^	0.0000^*^	0.0001^*^	0.0000^*^	0.01^*^	0.21	0.86	

*SD, standard deviation; min, minimum value; q1, first quartile; q3, third quartile; max, maximum value*.

* *Statistically significant p-values below 0.05*.

**Figure 2:**
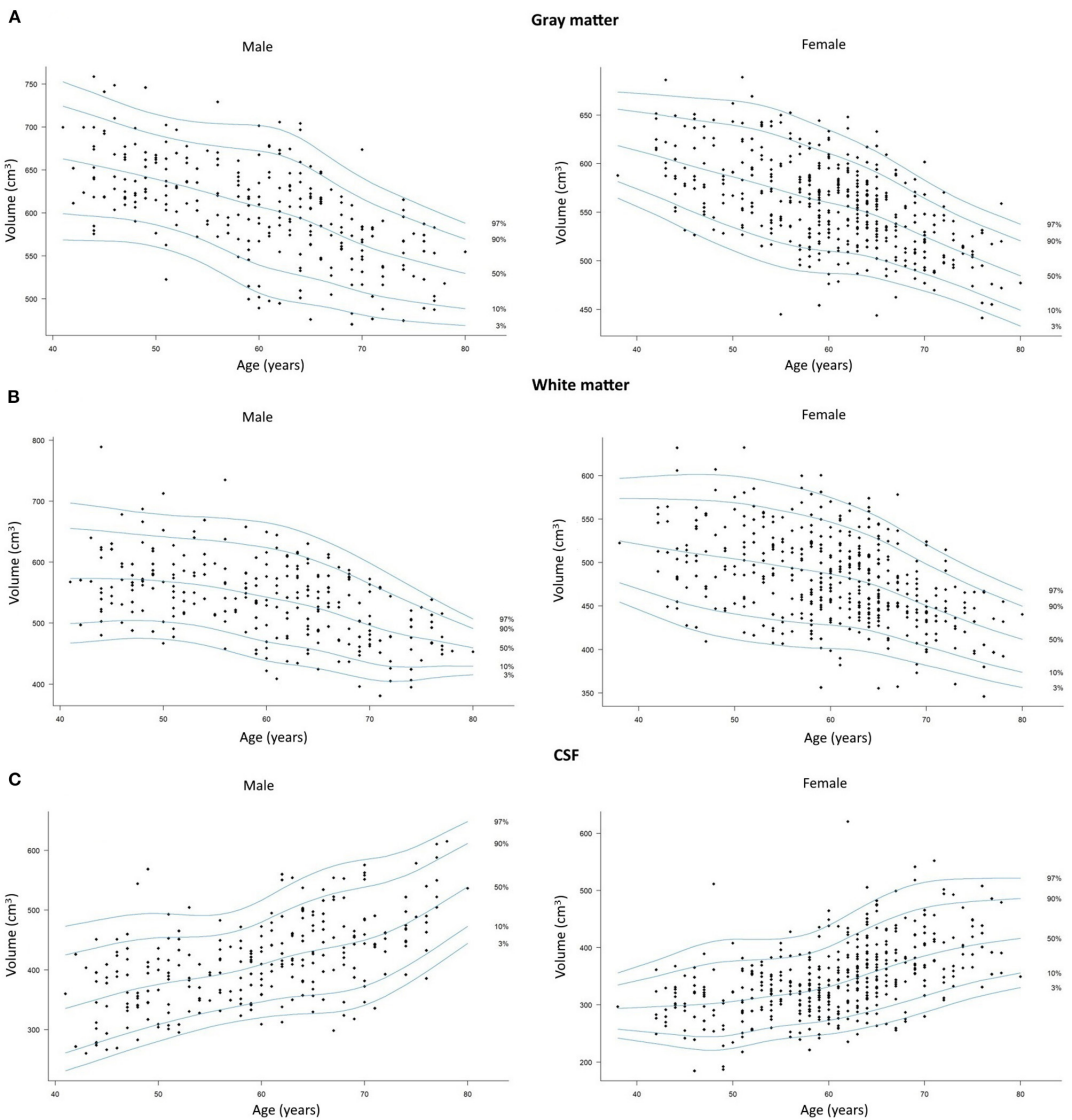
Scatterplots with percentile curves of the **(A)** total gray matter, **(B)** total white matter, and **(C)** cerebrospinal fluid volumes in men and women. Dots represent single subjects.

Figure 2 shows percentile curves for the total volumes of GM, WM, and CSF in different age groups.

Regarding cortical parameters, CT was found to significantly decrease with age in 58/148 (39%) cortical locations in women and in 53/148 (36%) locations in men (Supplementary Table 1). Age-related decrease in CT was found in the same 41 locations in both men and women within frontal, temporal, parietal, and occipital lobes (Table 5). In men, decreased CT was also found in 12 other locations within frontal, parietal, and occipital lobes as well as in the limbic lobe and the left occipitotemporal region. On the other hand, in women, decreased CT was seen in 17 other locations within paracentral lobules, right insula, as well as temporal, parietal, and occipital lobes (Table 5).

**Table 5:** Detailed list of locations showing significant changes during aging in both sexes and only in men or women.

**Parameter**	**In both sexes**	**Only in men**	**Only in women**
Cortical thickness	Left transverse frontopolar gyrus, opercular and triangular parts of both inferior frontal gyri, both middle frontal gyri, both superior frontal gyri, left lingual gyrus, right parahippocampal gyrus, right angular gyrus, right supramarginal gyrus, both superior parietal lobules, both post- and pre-central gyri, left precuneus, bilateral cuneus, both anterior transverse temporal gyri (of Heschl), lateral aspects and planum polare and planum tempolare of both superior temporal gyri, both middle temporal gyri, left central sulcus, inferior segments of circular sulcus of both insulae, both superior frontal sulci, right intraparietal and transverse parietal sulci and both post-central sulci	Right transverse frontopolar gyrus, anterior part of the right middle cingulate gyrus, the orbital part of the left frontal gyrus, left superior occipital gyrus, left fusiform gyrus, left parahippocampal gyrus, left angular gyrus, left supramarginal gyrus, posterior ramus of the left lateral fissure, right central sulcus, marginal branch of the right cingulate sulcus, left intraparietal and transverse parietal sulci	Both paracentral lobules, right long insular gyrus, right superior occipital gyrus, right lingual gyrus, both occipital poles, both temporal poles, both calcarine sulci, inferior and superior parts of precentral sulci and both transverse temporal sulci
Sulcal depth	Planum polare of the right superior temporal gyrus and anterior segment of the circular sulcus of the right insula		Right subcallosal gyrus, both anterior transverse temporal gyri (of Heschl), planum polare of the left superior temporal gyrus, horizontal ramus of the anterior aspect of the right transverse fissure and vertical ramus of the anterior aspect of the left transverse fissure, posterior ramus of the left lateral fissure, right temporal pole, anterior segment of the circular sulcus of the left insula, inferior and superior segments of the circular sulci of both insulae, left anterior transverse collateral sulcus, both lingual sulci and both transverse temporal sulci
Gyrification index	Posterior ramus of the right lateral fissure and inferior segments of circular sulci of both insulae		The left supramarginal gyrus and superior segment of the circular sulcus of the left insula
Fractional dimension	Superior segment of the circular sulcus of the left insula and left short insular gyrus	Left subcentral gyrus	Isthmus of the left cingulate gyrus and left post-central sulcus

**Figure 3:**
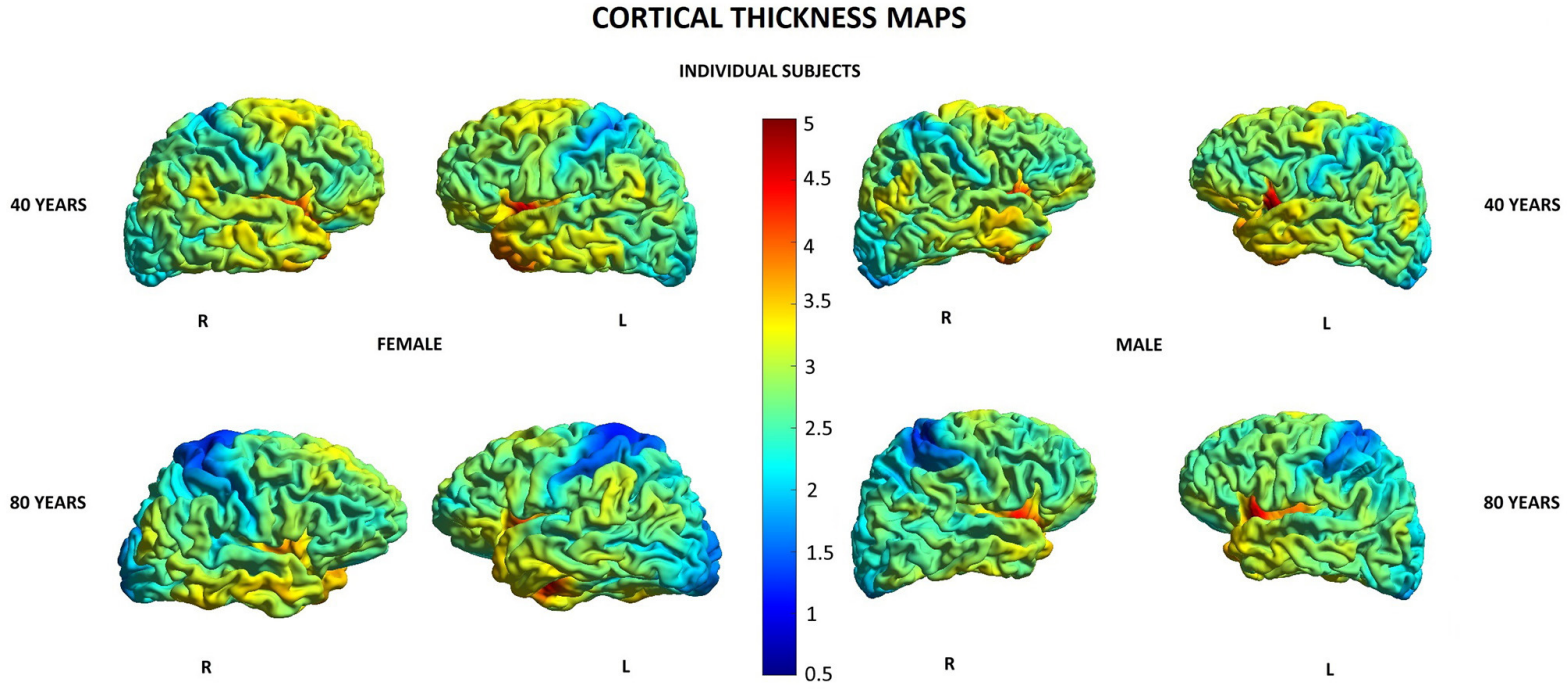
Cortical maps of age-related distribution of cortical thickness in 40- and 80-year-old female and male individual subjects. Lateral views of right (R) and left (L) hemispheres.

**Figure 4:**
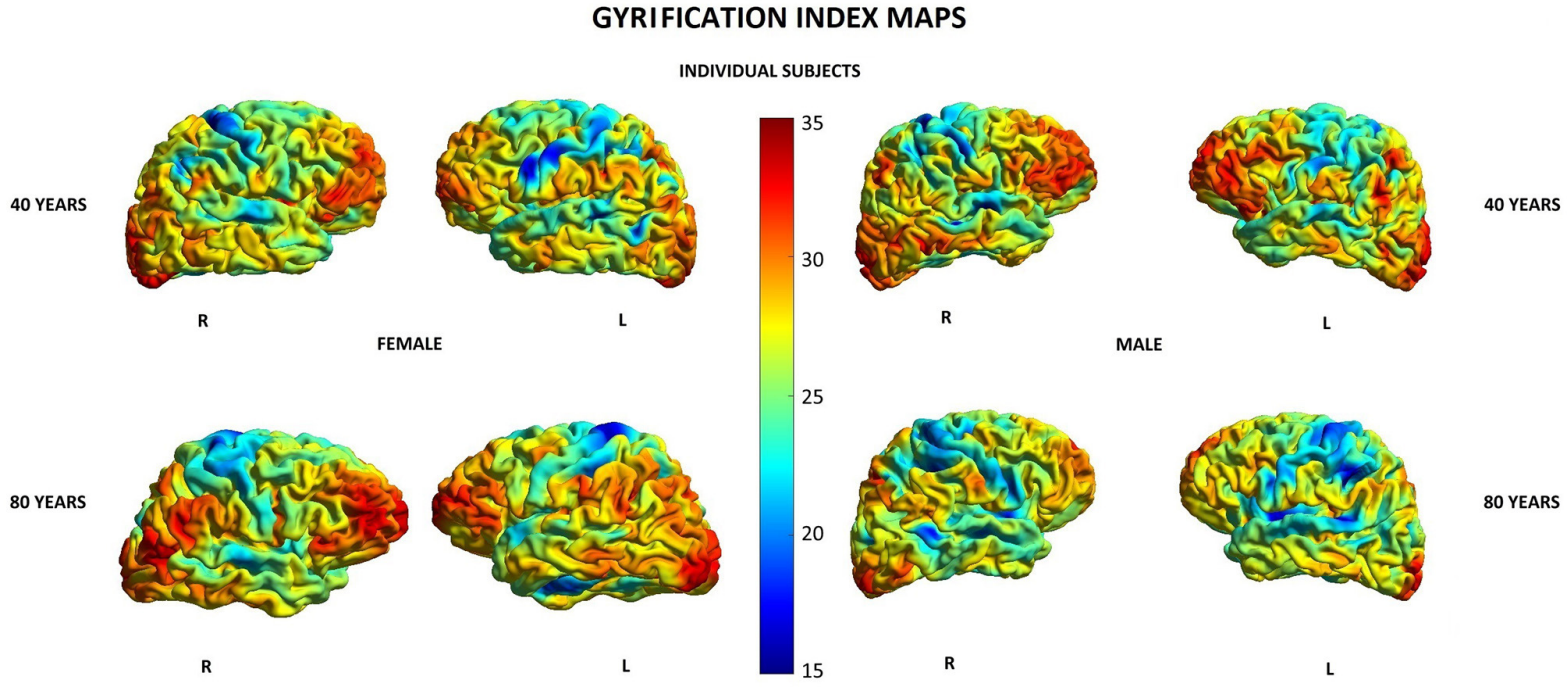
Cortical maps of age-related distribution of gyrification index in 40- and 80-year-old female and male individual subjects. Lateral views of right (R) and left (L) hemispheres.

**Figure 5:**
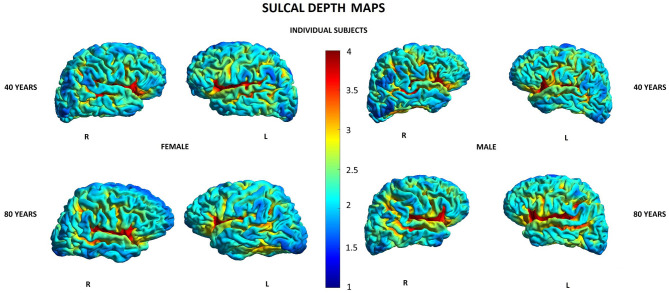
Cortical maps of age-related distribution of sulcal depth in 40- and 80-year-old female and male individual subjects. Lateral views of right (R) and left (L) hemispheres.

**Figure 6:**
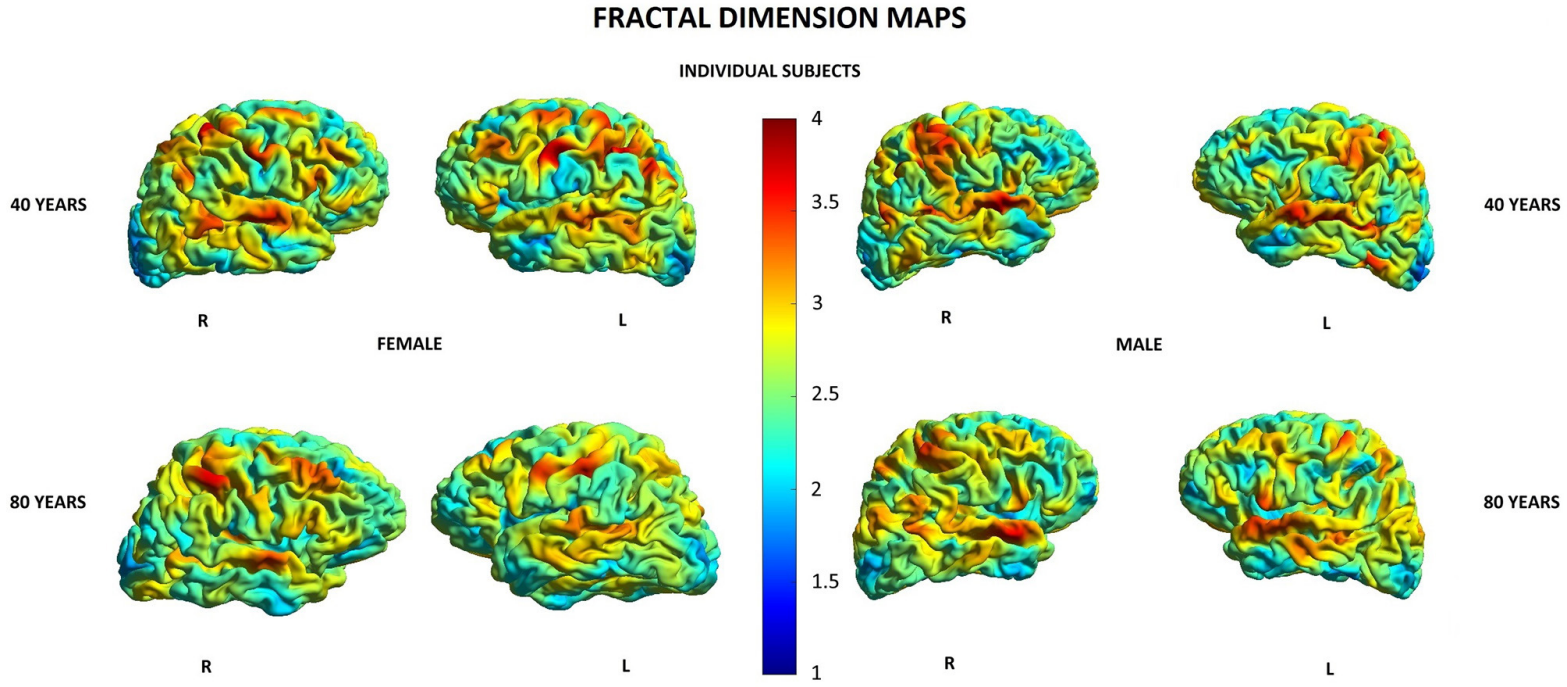
Cortical maps of age-related distribution of fractional dimension in 40- and 80-year-old female and male individual subjects. Lateral views of right (R) and left (L) hemispheres.

Other parameters showed less changes during aging: SD was found to increase with age in 20/148 (13.5%) locations in women and in 2/148 (1.3%) locations in men, GI was lowered in 5/148 (3.4%) locations in women and in 3/148 (2.0%) locations in men, and FD was decreased in 4/148 (2.7%) locations in women compared to 3/148 (2.0%) locations in men (Supplementary Table 1). In both men and women, SD increased with age in two locations within the right temporal lobe and right insula, while in women, additionally, also in other 18 locations within structures of the limbic, temporal, and parietal lobes as well as the left insula (Table 5). In both men and women, GI was lower with age in three locations including the right lateral fissure and insular sulci bilaterally, while in women, this parameter was also changed in two other locations within the parietal lobe and insula on the left side (Table 5). In both men and women, FD decreased with aging in the same two locations within the left insula. In men, FD was additionally changed in the left subcentral gyrus, while in women, in the left cingulate gyrus and left post-central sulcus (Table 5). Figures 3–6 show 3D color visualizations of age-related distributions of CT, GI, SD, and FD in a 40- and 80-year-old male and female individual subjects.

## Discussion

The aim of our study was to analyze age-related atrophy of GM and WM using VBM as well as to evaluate in detail cortical aging separately in men and women using several parameters derived from SBM analysis in order to find patterns of sex-specific aging of the brain.

In the literature, there are several reports on sex-specific aging of brain structures evaluated mainly with MR volumetry but their results are very inconsistent. Some of these studies have reported age–sex relations in volumes of certain brain structures (33–39), while others have found no such interactions (40). All these studies have been performed by comparing brain volumes between age-matched female and male groups without focusing on differences in aging patterns between both sexes, as it is done in our study, so the results are difficult to compare. Reports on different patterns of brain aging in both sexes are scarce in the literature. Brouwer et al. in the study of adolescents (age 9–23 years) on the basis of structural MR imaging reported similar aging/developmental patterns in both sexes, although female individuals were found to pass through this process, on average, 1 year earlier. According to the authors, the difference between brain age and chronological age was heritable (41). Contradictory results were reported by Goyal et al. who, using PET studies, estimated brain metabolism in men and women and found women's brains to be, on average, 3.8 years younger and men's brains 2.4 years older than their chronological ages, concluding that women have lower metabolic age compared to men (26).

In our study, similarly to previous reports, we found volumes of total GM, WM, and CSF lower in women compared to men, which can be explained by and overall smaller size of the female head and brain compared to men (21, 24). In both sexes, we found loss of GM and WM volumes as well as an increase in the CSF volume to begin below the age of 45 years and to gradually progress till the age of 80, but the dynamics of these processes were found to be different in each sex. Our results showed that compared to men, the female cortex starts to age approximately 5 years earlier around 51–55 years of age, while the WM is spared from atrophy for a longer period of time till the age of 61–65. In female brains, a dynamic WM loss was found to start 10 years later than GM atrophy and lasted 5 years longer than rapid GM changes. On the other hand, in men, GM and WM atrophy was found to progress in a fairly parallel way in terms of their initiation, dynamics, and termination. In our study, due to ROI-based approach, we were able to obtain parametric results of the evaluated parameters and provide percentile curves for total GM, WM, and CSF that can be used as sources of normative values for single subjects (Figure 2).

In the second part of our study, we investigated changes in the cortical indices during aging. There have been several reports evaluating sex-related differences in those parameters between age-matched men and women but not their age-related patterns of changes across the lifespan. Im et al. reported significant localized cortical thickening in women in frontal, parietal, and occipital lobes (42), while other researchers found significant rightward asymmetry and greater gyrification in women in the frontal and parietal regions, which indicated increased complexity of the cortical surface area in women (43, 44). Forde et al. in a study on 3,069 participants, from 8 to 95 years of age, found widespread greater variability in male participants compared with female participants in the cortical surface area and global and subcortical volumes for discrete brain regions with similar variance in CT for male and female participants (45). On the other hand, a large meta-analysis of 16,683 healthy individuals aged 1–90 (47% female) performed by ENIGMA group showed similar results regarding cortical surface area and subcortical measures but not CT in which greater variability in male individuals was found in 60% of CT measures starting already in the childhood and being stable across the lifespan (46).

In our study, we did not focus on differences in brain parameters of cortical surface and complexity between age-matched men and women, but the main goal was to evaluate their changes over time. Evaluation of changes in different cortical indices during aging revealed CT to be the most affected parameter in both men and women. At the same time, this parameter was found to change in a very similar way in both sexes. In both sexes, similar percentage of brain structures showed cortical thinning during aging (39% in women and 36% in men), and in a great majority in both sexes, they regarded the same cortical locations in all cerebral lobes with additional involvement of the limbic lobe in men and the right insula in women. Such widespread age-related reductions in CT are in concordance with previous reports revealing age-related thinning across the majority of the cortex with the most robust changes regarding bilateral frontal cortices, superior temporal regions, and supramarginal gyri. These changes are believed to be related to a decrease in dendritic density of neurons as well as changes in cortical myelination (13).

In our study, SD, GI, and FD were changed during aging only in a small number of locations in men (1.3, 2.0, and 2.0%, respectively), while those numbers were significantly higher in women (13.5, 3.4, and 2.7%, respectively). These results show that the process of cortical aging is more complex in the female brain, affecting not only volume and thickness but also other parameters reflecting cortical organization and complexity. The relations between these cortical parameters as well as clinical implications of their changes are not well understood yet. Several studies have revealed that age-related decreases in GI show different topography than changes in CT and are more related to the parameter of SD (14–16). Furthermore, SD, GI, and FD have been reported to show positive associations with cognitive function (15, 47, 48). To our knowledge, there are no other reports on the age–sex-related patterns of changes in these parameters across the lifespan.

The methods of post-processing used in our study were based on RBM calculated using CAT12 and SPM12 software. CAT12 software is a new toolbox that is comparable with FreeSurfer in terms of accuracy. A study by Seiger et al. provided evidence that CAT12 delivers accurate and robust CT estimates and can be considered a fast and reliable alternative to FreeSurfer. Its main advantage is the processing time, which is significantly reduced per subject (2). In our study, we performed RBM, which, in regard to classic VBM, gives unmodulated values for different parameters obtained arbitrary in predefined brain locations. These parametric data may be used directly when referencing to other studies and evaluating single subjects. In comparison, computation in voxel-wise manner does not allow for single subject's assessments.

To assess cortical thickness and complexity, we used SBM, which, due to the sheet-like topology of the cortex, seems to be more appropriate than classical VBM coordinate systems. SBM provides better intersubject averaging and allows the development of tools for automatically parcellating the cortex in a reproducible and accurate way. Surface-based cortical labeling methods have major advantages as compared to VBM. The complex folded anatomy of the human cerebral cortex is visually simplified by the inflation process. Second, interindividual differences in cortical anatomy are better depicted in SBM compared to that in VBM approaches. It also respects cortical topology especially in regard to points with close surface coordinates (32). One major limitation to this surface atlas is that it only labels the cortex, ignoring subcortical structures. Nevertheless, in the FreeSurfer reconstruction stream, deep structures are labeled by a volume-based tool using a similar probabilistic algorithm resulting in the labeling of cortical as well as subcortical and ventricular structures at the end of the process, which was also done in our study (32).

The main strengths of our study are the large number of subjects and the use of multiple subtle cortical measures assessed with the use of the same scanning protocol and methodology based on both VBM and SBM with an RBM approach. Our measurements obtained with RBM allowed us for a detailed quantification of the assessed parameters and providing percentile curves for total GM, WM, and CSF volumes, which are a unique display of population characteristics. These data may be used by other researchers studying different populations to compare temporal trajectories of brain aging. Furthermore, for SBM, we used parcellations available in Destrieux Atlas, which allows for the valuation of 150 cortical locations. Other commonly used atlases like, for example, Desikan–Killiany, enable analysis of significantly smaller number of cortical locations.

The limitation of our study is that it was conducted on a 1.5-T MR scanner, although it has to be stressed that several important recent studies on brain volumetry and cortical measures that were cited in this paper were also conducted using 1.5-T scanners. Another limitation due to time constrains was the lack of advanced functional imaging methods such as diffusion, perfusion, or resting-state functional MRI (fMRI), which could bring other data than only structural information. Another weakness of the research is its cross-sectional design and the study sample that consisted of a single population of Central Europe Caucasians and thus should be treated as population specific. However, it has to be stressed that up until now, there have not been any detailed studies on differences in brain morphology, volumetry, and brain aging in different ethnic population and with regard to sex dimorphism.

## Conclusions

Male and female brains start aging at the similar age of below 45. The process of aging is not the same in both sexes. Compared to men, in women, the cortex is affected earlier and in a more complex pattern including not only the cortical loss but also other alterations within the cortical shape, while white matter seems to be spared from atrophy for a longer period of time. In men, GM and WM loss as well as a CSF increase are processes parallel in time regarding their initiation, dynamics, and termination, while in women, there is a time gap of approximately 10 years between the initiation of dynamic cortical and white matter atrophy. Different patterns of brain aging in men and women may explain different vulnerability of both sexes to degenerative brain diseases and cognitive impairment, but further research is needed to fully understand their relations with clinical outcome. We think that being aware of differences in age-related brain changes between men and women makes it very important for next studies on this topic or on degenerative diseases. It shows the importance of separate evaluation of male and female sexes in the context of brain aging and degeneration.

## Data Availability Statement

The original contributions presented in the study are included in the article/Supplementary Material, further inquiries can be directed to the corresponding author.

## Ethics Statement

The studies involving human participants were reviewed and approved by the Wroclaw Medical University Ethics Committee for conducting research involving humans. The patients/participants provided their written informed consent to participate in this study.

## Author Contributions

PP conducted all volumetric measurements, analysed and interpreted data, conducted literature search, and wrote the manuscript. JB and MS contributed to the study design and critically reviewed the manuscript. AZ contributed to the study design, supervised writing of the manuscript, critically reviewed the paper, and supervised the research project. All authors contributed to the article and approved the submitted version.

## Conflict of Interest

The authors declare that the research was conducted in the absence of any commercial or financial relationships that could be construed as a potential conflict of interest.
